# Origin and evolution of the enhancer of split complex

**DOI:** 10.1186/s12864-015-1926-1

**Published:** 2015-09-18

**Authors:** Peter K. Dearden

**Affiliations:** Genetics Otago and Gravida (National Centre for Growth and Development), Biochemistry Department, University of Otago, Dunedin, Aotearoa New Zealand

## Abstract

**Background:**

The Enhancer of split complex is an unusual gene complex found in Arthropod genomes. Where known this complex of genes is often regulated by Notch cell signalling and is critically important for neurogenesis. The Enhancer of split complex is made up of two different classes of genes, basic helix-loop-helix-orange domain transcription factors and bearded class genes. The association of these genes has been detected in the genomes of insects and crustaceans.

**Results:**

Tracing the evolution of the Enhancer of split complex in recently sequenced Arthropod genomes indicates that enhancer of split basic helix-loop-helix orange domain genes arose before the common ancestor of insects and Crustacea, and before the formation of the complex. Throughout insect and crustacean evolution, a four-gene cluster has been present with lineage specific gene losses and duplications. The complex can be found in the vast majority of genomes, but appears to be missing from the genomes of chalcid wasps, raising questions as to how they carry out neurogenesis in the absence of these crucial genes.

**Conclusions:**

The enhancer of split complex arose in the common ancestor of Crustacea and insects, probably through the linkage of a basic helix-loop-helix orange domain gene and a bearded class gene. The complex has been maintained, with variations, throughout insect and crustacean evolution indicating some function of the complex, such as coordinate regulation, may maintain its structure through evolutionary time.

**Electronic supplementary material:**

The online version of this article (doi:10.1186/s12864-015-1926-1) contains supplementary material, which is available to authorized users.

## Background

Evolutionary conserved complexes of genes are rare in insect genomes [1] while relatively common in vertebrates [2–4]. The best characterised is the Hox complex, found in the genomes of widely diverse animals, in which interlinked and coordinate gene expression appear to stabilise the genomic structure of the complex over evolutionary time [5]. Complexes of genes that remain intact over wide evolutionary distances in insect genomes presumably have similar features to the Hox complex, such as coordinate regulation, that maintain their genomic structure, while the genome is rearranged around them.

The Enhancer of Split Complex (E(spl)-C) is an unusual and conserved complex of genes first identified in *Drosophila melanogaster*. This complex differs from most in that the genes it contains encode two completely different sorts of proteins: bHLH-Orange domain transcription factors [6, 7] (bHLHO), and Bearded class proteins [8, 9] (Brd). The association between these two types of genes has been found in the genomes of both insects and crustaceans [10], implying that this complex first formed through the association of these genes rather than the more usual gene duplication.

A limited survey of insect and crustacean genomes has shown that the E(spl) complex is ancestrally made up of three bHLHO encoding genes (*bHLH1*, *bHLH2* and *her*) and a single Brd-class gene [10]. The structure of the complex is modified in some insects, *Drosophila* being an example, where two bHLH-orange domain genes are absent from the complex and there are seven copies of the remaining one, the Brd-class gene, *mα*, has been duplicated and there are a range of unrelated genes inserted in the complex [6, 9–16].

The E(spl)-C was first identified as a modifier of the *Notch* mutant *Split* [17]. Subsequent studies have shown that the bHLH proteins encoded by E(spl)-C act as effectors of Notch cell signalling [13, 18]. During neurogenesis in *Drosophila*, presumptive neuroblasts signal to surrounding cells in proneural clusters through the Notch cell-signalling pathway (Reviewed in [19–21]). This pathway leads to expression of E(spl)-C bHLH genes [18], which act to repress the expression [22], and function [23], of proneural genes, a set of transcription factors that promote neural cell fate [24, 25]. Presumptive neuroblasts thus signal surrounding cells to block their differentiation as neuroblasts through the activation of Notch cell signalling, and the expression of the E(spl) bHLHO domain proteins [26–29]. E(spl) bHLHO domain proteins encode a c-terminal WRPW motif which recruits the transcriptional repressor Groucho, which in turn acts to attenuate gene expression by promoting RNA polymerase pausing, and causes local histone deacetylation [30]. E(spl) bHLHO domain proteins thus target transcriptional repression to proneural genes, and proneural gene targets.

Brd class proteins from the E(spl)-C, in contrast, antagonise Notch signalling by interacting with Neuralised [31], an E3 ubiquitin ligase, stimulating the degradation of a Notch ligand, Delta [32]. In *Drosophila*, brd-class proteins particularly act to pattern adult sensory precursor formation [8, 9]. Bearded class genes encode small proteins with amphipathic alpha helices, and little sequence conservation [9].

The genes contained within the E(spl)-C are regulated in a range of ways. While individual enhancer elements for some of the genes have been identified [33, 34], the entire complex appears also to be activated by Su(H) [9, 35–40], a transcription factor usually regulated by Notch signalling [41]. This Notch responsiveness is also found in a crustacean, *Daphnia* [42]. Individual transcripts are repressed, in *Drosophila*, by miRNAs binding to 3′UTR located sites (named GY, Brd and K- boxes) [15, 43, 44]. There is also evidence for coordinate regulation of the E(Spl)-C by cohesin [45], a chromatin structure regulating protein that coats the E(spl)-C in cells and represses expression, and repression by Polycomb group proteins [46]. The genomics DNA containing the E(spl)-C also is structured in three dimensions in cells such that the chromatin of the complex interacts with itself forming an isolated domain, but does not interact with flanking regions [46]. This self-interactive structure implies the complex is regulated in a coordinated manner [46].

E(Spl)-C bHLH proteins are closely related to other clades of insect bHLH-orange domain proteins, including *clockwork orange* (*cwo*), *Similar to Deadpa*n, (*Side*), *Hairy andE(spl) related with aYRPW domain* (*Hey*), *hairy* (*h*), *deadpan* (*dpn*). These genes have multiple roles in insects. *Hairy* is a regulator of segmentation [47, 48], acting as a pair-rule gene in many arthropods [49–51]. *Deadpan* [52] has multiple roles in *Drosophila* development, such as dosage compensation [53] and regulating neuroblast proliferation through responding to Notch signalling [54]. *Hey* has been shown to regulate neuron fate determination [55]. *Cwo* (clockwork orange) has a role in regulating the circadian clock [56–59]. *Side* has no identified function in *Drosophila*. In non-arthropod animals, closely related genes, the HES genes (hairy-enhancer of split genes) act, in most situations, as effectors of Notch signalling [60, 61] though some these genes do have non-Notch related roles [62, 63]. Given this close phylogenetic relationship, and the role in Notch signalling conserved between arthropods and vertebrates, it seems likely that Notch responsiveness, and a role as an effector of Notch signalling, may be an ancestral function for this group of transcription factors. Indeed expression of *E(spl)-mδ* produces a neurogenic effect when mis-expressed in *Xenopus* embryos [64].

The unusual nature of the E(spl)-C, containing two types of genes, and its potential coordinate regulation, make understanding the dynamics of its evolution important. Here I examine the structure and relationships of the E(spl)-C from arthropod and onychophoran genomes, particularly those provided by the i5K consortium [65, 66]. The i5K consortium provides high quality Arthropod genome sequences that allow both the identification of genes, and examination of genome structure. This dataset allows us to trace both the origins and subsequent evolution of the E(spl)-C in arthropods.

## Results and discussion

### Phylogeny of all isolated E(spl) bHLH genes recovers three major clades

**Fig. 1:**
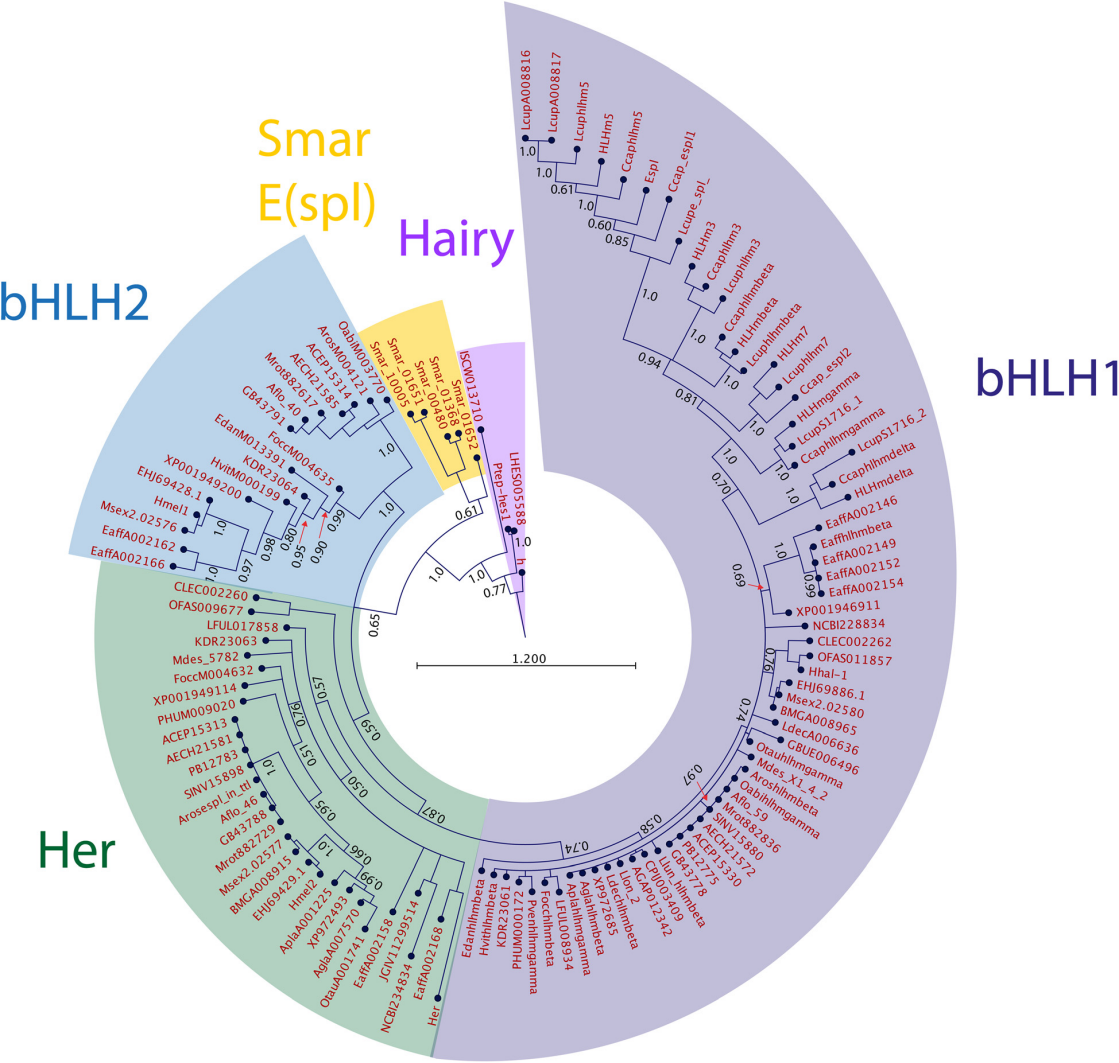
Relationships of E(Spl) bHLH genes. Bayesian Phylogram of E(spl)-C related bHLH proteins generated using WAG model of amino-acid evolution. Clade of bHLH proteins are marked by coloured areas and named. Node labels indicate posterior probabilities. The tree is rooted with hairy sequences from insects and chelicerates. Protein identifiers refer to names in Additional file 1: Table S1

Searching for E(spl)-like bHLH sequences in arthropod genomes identifies large numbers of sequences with similarity to E(spl) bHLHs, and other clades of bHLHO domain sequences. In most species of insects and Crustacea, multiple bHLHO sequences can be identified with strong similarity to E(Spl)-C bHLHs, and these genes are often found adjacent to each other in contigs, linkage groups or genome scaffolds. Aligning the protein products of these genes and reconstructing their relationships using Bayesian phylogenetics [67] identifies the three major clades of E(spl) bHLHO proteins as first described in Duncan and Dearden [10] (her, bHLH1 and bHLH2) (Fig. 1). The analysis also indicates that the *Strigamia maritima* (centipede) genome contains a number E(Spl) bHLH proteins, but these do not fall into the three classes of E(spl) bHLH proteins found in crustacean and insects. Orthologues of non E(Spl) bHLHO proteins robustly fall outside the E(Spl) bHLH clades (Protein identifiers refer to names in Additional file 1: Table S1).

Branch-lengths within the E(spl) bHLH clades are short, indicating strong conservation of sequence, but these branch lengths increase between bHLH1 proteins from Diptera, implying more sequence divergence in that group. The assignment of E(spl) bHLH genes from crustacean and insect genomes to these three clades of allows us to interpret the genomic structure of the E(spl)-C in crustacean and insect genomes.

### Genomic analysis of E(spl) genes indicates clusters and conserved structure

**Fig. 2:**
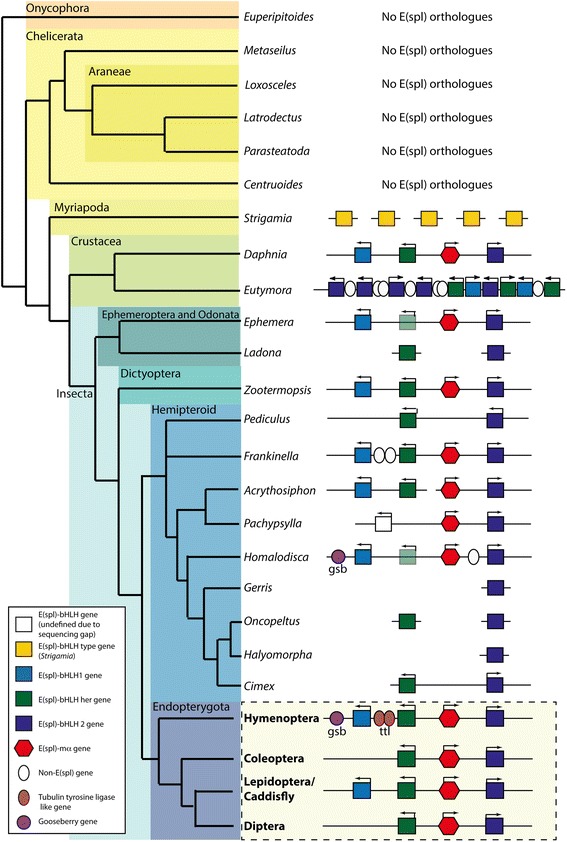
Genomic Structures of E(spl) complexes. Schematic phylogeny of Arthropods with structures of E(spl)-C from examined species. Colour coding of bHLH genes follows Fig. 1 (*bHLH-1*, light blue; *bHLH2*, Dark blue; *her*, green). Red hexagons indicate *mα* genes. White ovals indicate inserted genes with no homology to bHLHO or *mα*, brown ovals represent *Tubulin Tyrosine ligase* genes. Purple circles mark *gooseberry* genes. Where the colour of a bHLH gene is lightened, identification of this gene is only through placement in the complex due to gaps in the genome sequence. Where a white square is shown, placement in the complex cannot indicate the identity of the partial gene sequence. Yellow squares indicate E(spl) type bHLHs from *Strigamia* that are not able to be classified. Arrows indicate direction of transcription. Structures for the Endopterygota are predicted based on analyses of complex from these groups (Figs. 3, 4, 5 and 6)

Duncan and Dearden [10] identified a four gene E(spl) complex as the ancestral state for insects and Crustacea. This four-gene cluster (*bHLH-2*, *her*, *mα* and *bHLH-1*) was identified in the genome of the crustacean *Daphnia* and in sequenced insect genomes [10]. I have expanded this analysis, using the i5k consortium data, to identify E(spl)-Cs within Arthropod and Onycophoran genomes and then used phylogenetic analyses (Fig. 1) to categorise those genes. This analysis indicates that the bHLH complex is a component of the vast majority of insect genomes, but there are clade and species-specific losses and expansions. In Chelicerates, and the partial genome of an onycophoran (Velvet worm, Non arthropod Ecdysozoan, basal to arthropods), we can find no evidence for bHLHO proteins that are orthologous (by reciprocal blast) to E(spl) bHLHO proteins (Fig. 2). A myriapod genome [68] (*Strigamia*) encodes 5 E(spl) proteins, but these are not co-located in the genome. In two crustacean genomes (*Daphnia* and *Eurytemora*), the E(spl)-C is more apparent. In *Daphnia* (Water Flea), as described previously, a four-gene complex is present, with an identifiable Brd-class gene, *mα*, embedded within it. *Mα* genes are often difficult to identify by Blast alone because their sequence evolves rapidly. In all species in which *mα* genes could be identified by tblastn [69] of the genome, they were found in the E(spl)-C except in *Drosophila*, where a complex of Brd-class genes lies outside the E(spl)-C [31]. Given the difficulties in identifying these genes, it is possible that other members of the Brd-class are present outside the E(spl)-C in other species.

In *Eurytemora*, a copepod crustacean, the E(spl)-C is modified with duplications of all three E(spl) bHLH genes (two *bHLH-1*, three *her* and 5 *bHLH2*). Alongside this, no *mα* gene can be identified.

In insects, a four-gene complex can also be identified in deeply branching clades (Fig. 2). In a mayfly (*Ephemera*) and a termite (*Zootermopsis*), a four-gene complex, identical to *Daphnia,* is present.

In the large hemipteroid clade of insects, examples of a four-gene complex are relatively common (*Frankliniella* (Western flower thrips), *Acrythosiphon* (Pea Aphid, though this is split between two contigs) [10], and *Homalodisca* (Glassy-winged sharpshooter)). Despite this conservation, gene-loss has occurred in some Hemipteroid lineage, with species such as *Cimex* (bedbug) and *Oncopeltus* (milkweed bug) having only two bHLH genes (*her* and *bHLH2*) and species such as *Gerris* (Waterstrider) and *Halyomorpha* (Brown marmorated stink bug) having a single E(spl) gene (*bHLH2*). The patterns of change in the complex imply that such losses are lineage or species specific, and that the selective pressure to maintain the full E(spl)-C is somewhat reduced in this assemblage. In species without the full complex of genes, *mα* is invariably missing, though its fast sequence evolution means that it could be located elsewhere in the genome.

**Fig. 3:**
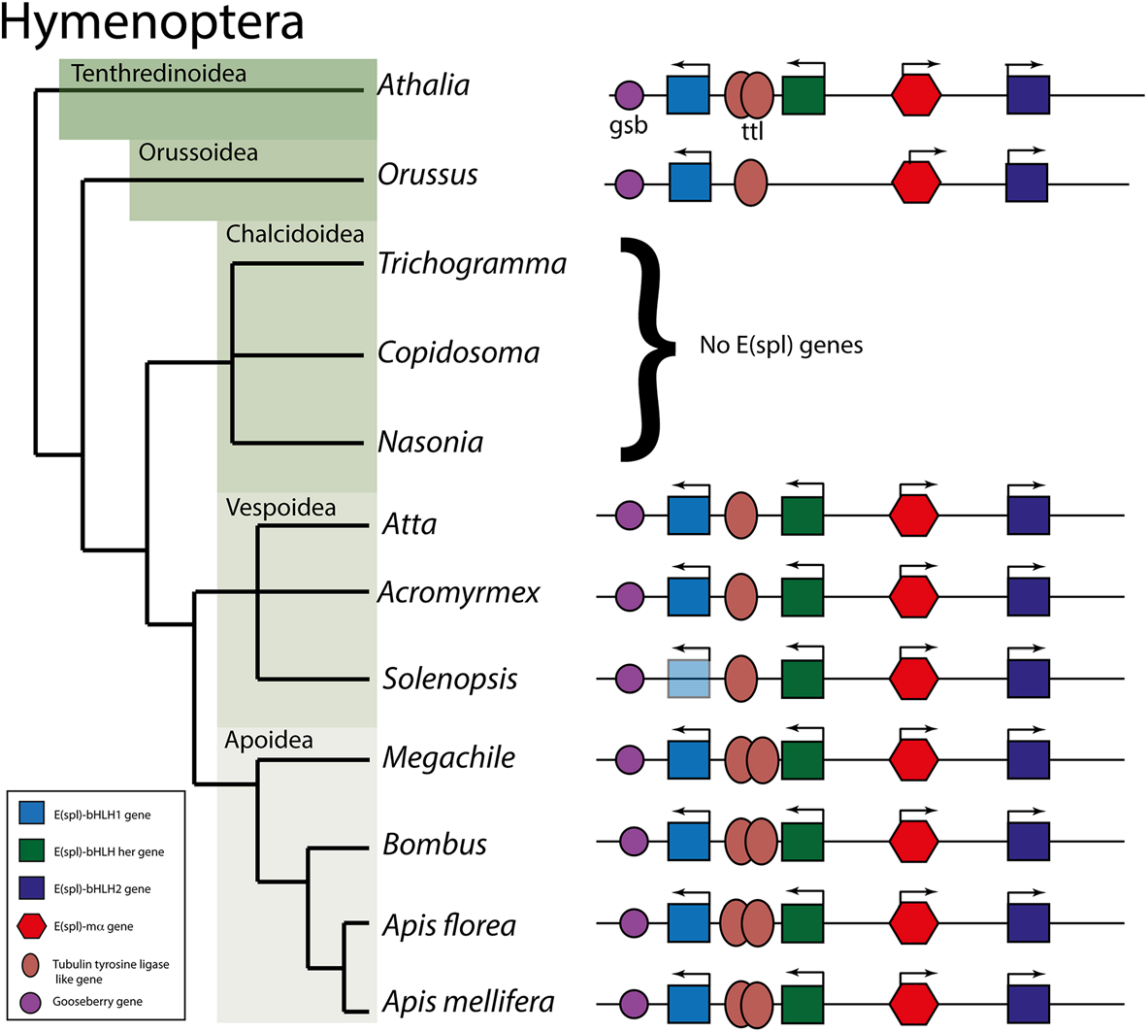
Genomic Structures of E(spl) complexes from Hymenoptera. Schematic phylogeny of Hymenoptera with structures of E(spl)-C from examined species. Colour coding of bHLH genes follows Fig. 1 (*bHLH-1*, light blue; *bHLH2*, Dark blue; *her*, green). Red hexagons indicate *mα*, genes. White ovals indicate inserted genes with no homology to bHLHO or *mα*, brown ovals represent *Tubulin Tyrosine ligase* genes. Purple circles mark *gooseberry* genes. Where the colour of a bHLH gene is lighter, identification of this gene is only through placement in the complex due to gaps in the genome sequence. Arrows indicate direction of transcription

Within Endopterygote lineages, the E(spl)-C appears more stable. In the Hymenoptera (Fig. 3), the classic four-gene complex is present in most species, though in *Orussus* (Parasitic woodwasp) one gene (*her*) is missing, and in Chalconid wasps (*Trichogramma*, *Copidosoma* and *Nasonia*) no complex is detectable (see later). With these exceptions the E(spl)-C (including flanking and unrelated genes inserted into the complex (see later)) is completely conserved in gene complement, order and orientation.

**Fig. 4:**
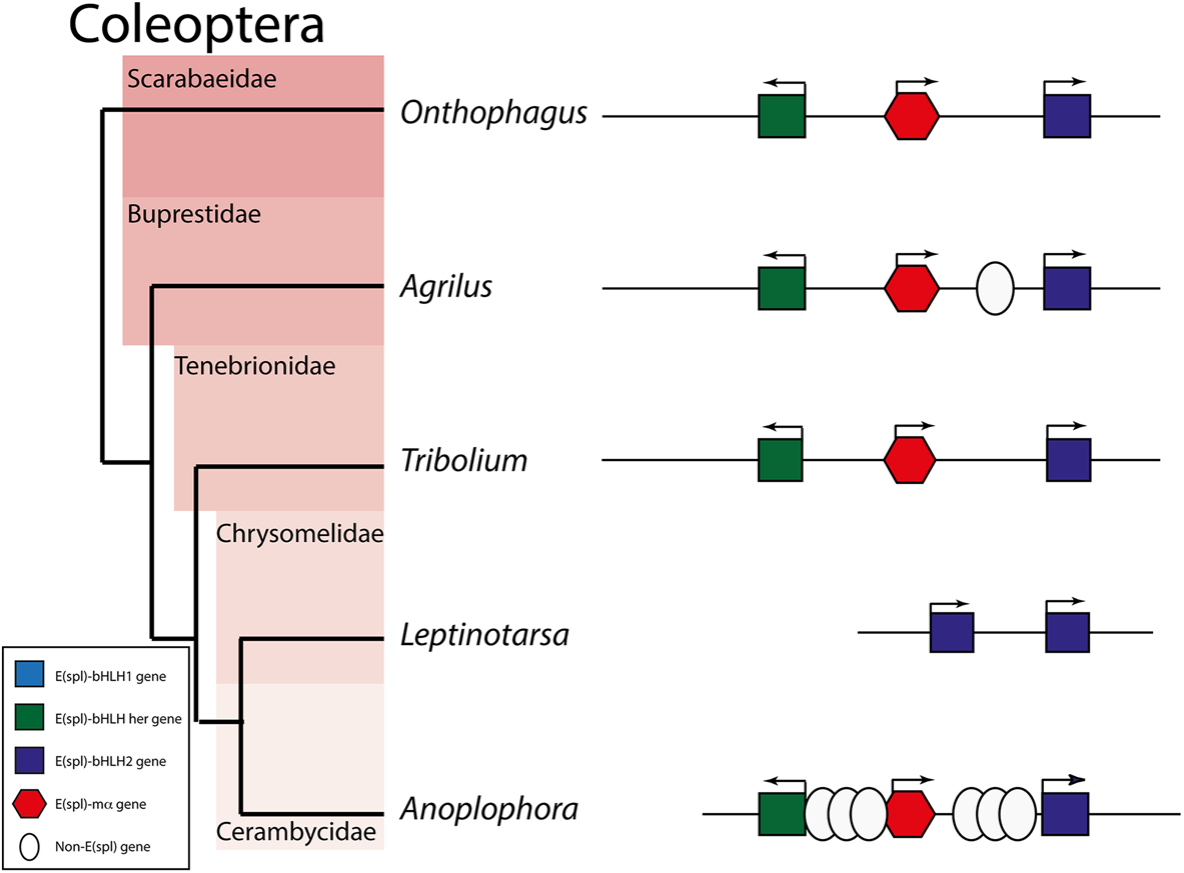
Genomic Structures of E(Spl) complexes from Coleoptera. Schematic phylogeny of Coleoptera with structures of E(spl)-C from examined species. Colour coding of bHLH genes follows Fig. 1 (*bHLH-1*, light blue; *bHLH2*, Dark blue; *her*, green). Red hexagons indicate *mα*, genes. White ovals indicate inserted genes with no homology to bHLHO or *mα*. Arrows indicate direction of transcription

In Coleoptera, by contrast, the complex is stable, but only contains three genes (*her*, *mα* and *bHLH2*) (Fig. 4). An orthologue of *bHLH1* is not present in any of the coleopteran genome examined. In *Leptinotarsa* (Colorado potato beetle), *her* and *mα* are missing, and *bHLH2* has been duplicated. The fact that *bHLH1* is absent from all Coleopteran species examined indicates that it was likely lost early in Coleopteran evolution.

**Fig. 5:**
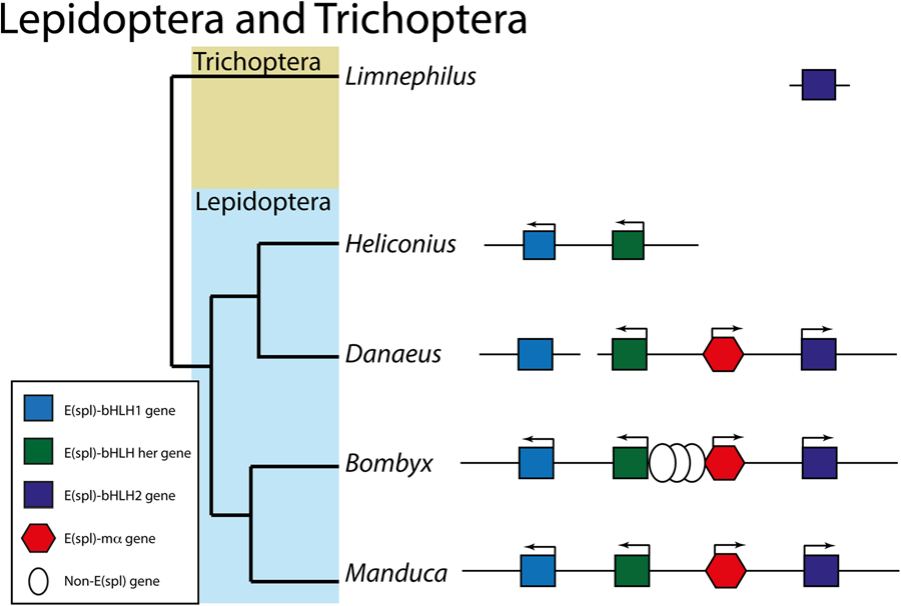
Genomic Structures of E(spl) complexes from Lepidoptera and Trichoptera. Schematic phylogeny of Trichoptera and Lepidoptera with structures of E(spl)-C from examined species. Colour coding of bHLH genes follows Fig. 1 (*bHLH-1*, light blue; *bHLH2*, Dark blue; *her*, green). Red hexagons indicate *mα*, genes. White ovals indicate inserted genes with no homology to bHLHO or *mα*. Arrows indicate direction of transcription

The basal four-gene complex can be found in the genomes of the Lepidopterans (Fig. 5) *Manduca* (Tobacco Hornworm*)*, and *Bombyx* [10] (Silk moth), and possibly in *Danaeus* (Monarch Butterfly) though in this genome the complex is split across two contigs. The complex is reduced to two genes (*bHLH1* and *her*) in *Heliconius* (Postman Butterfly). This is the only insect species of the 42 studied that does not have a *bHLH2* gene, raising the possibility that this may be a genome sequencing error. In a species of Caddisfly (*Limnephilus*, sister group to the Lepidoptera) only *bHLH2* can be found. It is unclear if this is species specific or lost in the entire lineage.

**Fig. 6:**
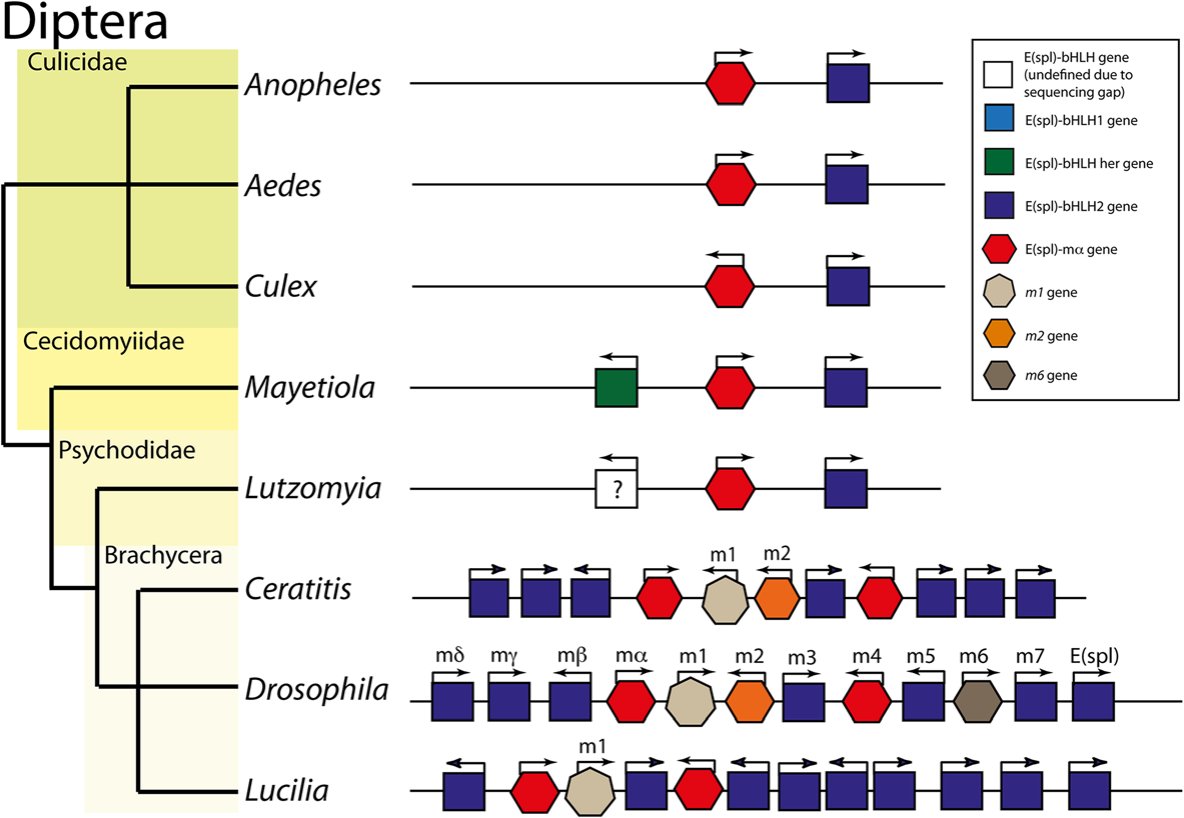
Schematic phylogeny of Diptera with structures of E(spl)-C from examined species. Colour coding of bHLH genes follows Fig. 1 (*bHLH-1*, light blue; *bHLH2*, Dark blue; *her*, green). Red hexagons indicate *mα*, genes. Orange hexagons indicate m2 genes related to *mα*. Light brown heptagons represent *m1* genes, dark brown hexagons *m6* genes. Arrows indicate direction of transcription

In Diptera (Fig. 6), *bHLH1* is absent from all genomes examined. In the Cuculidae (*Anopheles*, *Aedes* and *Culex*), *her* is also absent from the genome and *mα* and *bHLH2* make up the complex. In the Brachycera, the complex is expanded, with multiple copies of bHLH2; 7 in *Ceratitis* (Medfly), 7 in *Drosophila* species and 9 in *Lucilia* (Common Green Bottle fly). *Mα* is also expanded, with two copies in each genome. In *Drosophila*, *her* is present in the genome, though not linked to the E(spl)-C. In *Lucilia* and *Ceratitis*, no *her* ortholog is present.

*Her* is present in the E(spl)-C of *Mayetiola* (Hessian fly), a more deeply branching fly, and there is an unidentifiable gene (due to a gap in genome sequence), in the same position in the fragmentary genome of *Lutzomyia* (Blackfly). These patterns imply that at least a three gene complex (*her*, *mα* and *bHLH2*) was present in the common ancestor of Diptera, but that gene loss (Culicidae) and expansion of the complex (Brachycera) have extensively modified the E(spl)-C in this group.

In most of the complexes I have identified, the orientation of genes with respect to the direction of transcription of *mα* is conserved. *bHLH-1* and *her* are transcribed on the opposite strand to *mα*, and *bHLH2* on the same strand. The only variations to this pattern are in Diptera (Fig. 6) where the multiple copies of bHLH2 in Brachycera are transcribed from either strand, and in *Culex* (Mosquito), where *mα* and *bHLH2* are transcribed from opposite strands.

These data imply that the origins of the E(spl)-C lie in the pan-crustacean clade. I can find no evidence for E(spl)-C bHLH genes in chelicerates (4 genomes), the most basally branching clade of arthropods, nor the onychophora, the closest non-arthropod ecdysozoan group. While this analysis is not conclusive as to the presence of these genes in onychophorans (due to the partial nature of the genome sequence), their absence in all chelicerates examined is best explained by absence of these genes in this lineage, but could plausibly be due to gene loss.

The patterns of conservation, gene loss and expansion indicate that the E(spl)-C has a history of conservation of an ancestral four-gene structure, with gene-loss in some lineages, usually not affecting *bHLH2*, and expansion via gene duplication in Brachyceran flies, and the copepod *Eurytemora*. These expansions are difficult to explain, as their patterns of expansion are different. In *Eurytemora*, all bHLH genes are expanded, while *mα* is missing. In Brachycera, *bHLH1* and *her* are missing from the complex, with an expansion of *bHLH2* and mα. Given there are many species with two, or only one class of E(Spl) gene, these expansions are not best explained as a way to replace missing members of the complex, but may be related to complexity of gene regulation, or pattern formation, required from the complex.

### Insertions into the E(spl)-C

In *Drosophila melanogaster* (Fig. 6), 2 non bHLHO/mα genes are found in the E(spl)-C. These genes, *m1*(encoding a Kazal-type protease inhibitor) and *m6* (encoding a protein with a Myelin proteolipid protein PLP), do not produce Notch signalling- like defects when mutant, though m6, like the other E(spl)-C genes, is regulated by Notch signalling [70]. The insertion of *m1* and *m6* in the complex are conserved in other Drosophilids [10, 71] but only *m1* is conserved in the Bracyceran flies *Ceratitis* and *Lucilia*. Flanking the *Drosophila* complex is another Notch related gene, *groucho*, which encodes a protein that interacts with E(spl)-bHLH genes to supress gene expression [30, 72]. This gene does not flank the E(spl)-C outside *Drosophila* species.

Unrelated genes are inserted into the E(spl)-C in many insect species but these insertions are most often not conserved between species. The exception to this are a set of tubulin tyrosine ligase genes inserted between *bHLH1* and *her* in Hymenopteran genomes (Fig. 3). One or two of these genes are present in this location in all Hymenopteran examined that have an E(spl)-C. The maintenance of this insertion over 250 million years of evolutionary time, and the expression pattern of one of these genes in Honeybees [10] implies that these tyrosine-tubulin ligase genes may be regulated by Notch signalling.

The stability of the E(spl)-C in hymenopteran genomes extends to flanking genes. All Hymenopteran complexes are also flanked by a gene named *gooseberry* (Fig. 3). *Gooseberry* is a paired-box containing transcription factor that has been shown to have roles in patterning the nervous system and cuticle in a number of insect species [73–79] including hymenoptera [80, 81]. This gene is also found flanking the E(spl)-C in *Homoladisca* (Fig. 2), implying the association of *gooseberry* and E(spl)-C may date from the common ancestor of Endopterygota and the hemipteroid assemblage.

### Chalcid Wasps have lost the E(spl) complex

**Fig. 7:**
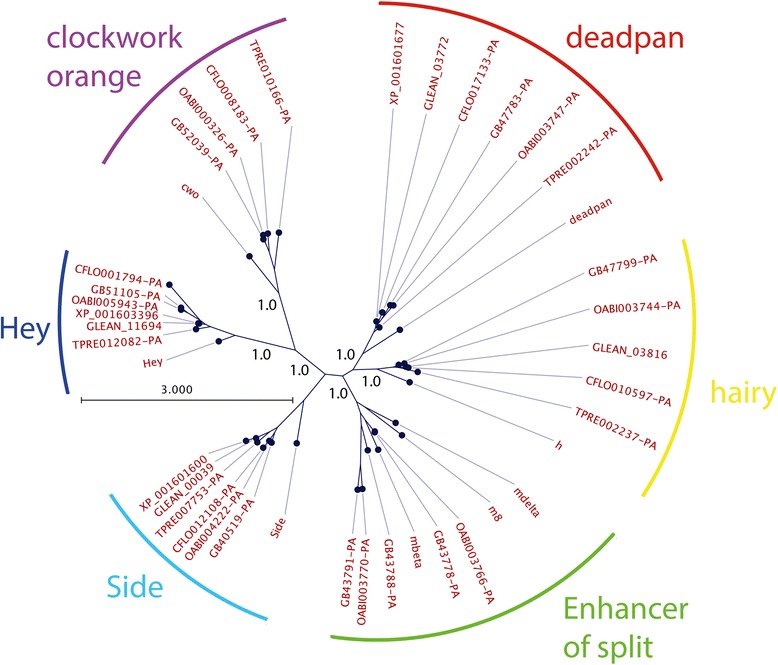
Relationships of bHLHO proteins from Chalconid wasps and other insects. Bayesian phylogram of representative hymenopteran bHLH-orange domain proteins reconstructed using the WAG model of protein evolution. While Chalconid wasps (genes identified by the prefixes, CFL (*Copidosoma*), TRE (*Trichogramma*) and XP_ or GLEAN (*Nasonia*)) have members of the hairy, deadpan, clockwork orange, Hey and Side families, they do not have proteins related to E(spl)-C bHLH proteins from *Apis* (GB identifiers, *Orussus* (woodwasp, OAB identifiers), or *Drosophila* (gene names)

While the E(spl)-C in the hymenoptera is highly conserved and stereotyped, I can find no evidence for E(spl)-C bHLHO genes in the genomes of three chalcid wasps. These species have a full complement of other bHLHO domain genes (Fig. 7), but no E(spl)-C orthologues are present in these genomes, either distributed, or in a complex. That this pattern of loss is present in three genomes, one of which is the well-sequenced (both genomes and transcriptomes) *Nasonia* genome, implies that loss of the complex in this group is not a sequencing error, but that Chalcid wasps have lost their E(spl)-C. These are the only group yet found in insects that do not have identifiable E(spl)-C genes. All of these wasp’s genomes encode the core components of the Notch signalling pathways, as well as other direct targets of Notch signalling (eg *glass*, *sugarless* etc.) (data not shown). This deficit is specific, therefore, to the genes of the E(spl)-C. That the E(spl)-C is missing from the genomes of these wasps raises important questions as to how cell specification in the nervous system of these animals is achieved.

Interestingly, studies of the effect of *Nasonia* venom on their fly hosts indicates that E(spl)-C genes are upregulated in the host in response to the venom [82], possibly to trigger developmental arrest. Is it possible that the evolution of resistance to their own venom has necessitated the deletion of the E(spl)-C from the genomes of wasps that use this mechanism?

### Origins of the e(spl) complex

**Fig. 8:**
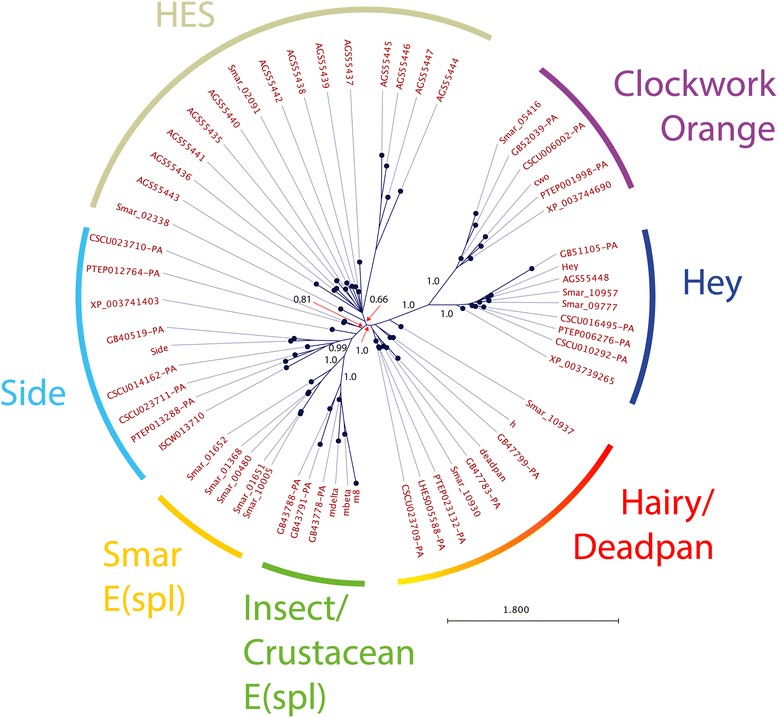
Relationships of bHLH Orange proteins from deeply branching arthropods and lophotrocozoan groups. Bayesian phylogram of representative bHLH-orange domain proteins reconstructed using the WAG model of protein evolution. bHLH Orange domain proteins from Chelicerates (PTEP, *Parasteatoda tepidariorum*; LHE, *Latrodectus hesperus*; ISCW, *Ixodes scapularis*; CSCU, *Centruroides exilicauda*; XP, *Metaseiulus occidentalis*) Insects (honeybee, GB; *Drosophila*; gene names) a myriapod, *Strigamia maritima* (smar_), and an Annelid, *Platynereis* (AGS identifiers) with well characterised HES genes [61]. *Strigamia* contains E(spl) related bHLHO proteins, but these form a separate clade to those in insects and Crustacea that make up the E(spl)-C

E(spl) bHLHO proteins are related to a broad family of BHLHO proteins found in animal genomes. In arthropods, 5 major families (Hairy/deadpan, Side, clockwork orange, hey and E(spl)) are present. These proteins are related to HES genes (Hairy/E(spl)-like proteins) found in Deuterostomes and Lophotrochozoan genomes [61]. I reconstructed the relationships between these genes using Bayesian techniques, focussing particularly on deep arthropod relationships, in order to understand the origins of E(spl)-C bHLHO proteins (Fig. 8). This analysis indicates that all of the 5 families of arthropod bHLHO proteins are related to the HES genes of other metazoa. Hairy/deadpan, side, cwo, hey and E(spl) are all equally related to the Notch regulated HES genes. E(spl) proteins are, however, restricted to Myriapods, Crustacea and Insects. The case of *Strigamia maritima* is an illuminating one. In this genome there are five genes encoding proteins closely related to E(spl)-C proteins, as well as examples of the other arthropod bHLHO (excepting Side). *Strigamia* also encodes two proteins similar to HES from Lophotrochozoa. Chelicerates (including 1 mite, 1 tick and two spiders) have a range of bHLHO proteins, but no E(Spl)-C related genes.

The E(spl)-like genes from *Strigamia* do not form a cluster in the genome, and the proteins encoded by these genes form a clade separate to the three crustacean/insect E(spl)-C bHLH clades. As most phylogenetic examinations of the placement of myriapods within the arthropods indicates that they are the sister group to crustaceans and insects, the origins of E(spl)-c bHLH proteins must lie somewhere after the separation of the lineage leading to chelicerates, but before the last common ancestor of insects/crustacea and myriapods. E(spl)-C bHLHO proteins thus pre-date the origin of the E(spl)-C, which is present only in the genomes of insects and crustacea.

These differences in the organisation of the E(spl)-C genes and complex mirror, to some extent, differences between these clades in the presence of the neural stem cells, neuroblasts, that the E(spl)-C regulates. In chelicerates and myriapods there is no evidence for cells similar to the neural stem cells that arise out of proneural clusters and repress their neighbours through Notch signalling and the E(spl)-C in *Drosophila* [83–87]. Neither of these groups have an E(spl)-C in our analyses, while Crustacea and insects, which do have identifiable neuroblasts, do. There is some evidence that Myriapods, which have no E(spl)-C but do have E(Spl) bHLHO proteins, may have specialised neural precursors in groups of cells specified to become neural [83]. Understanding how neural cells are specified in these groups, and how this is related to Notch signalling, will allow us to determine if the formation of the E(spl)-C is linked to the evolution of neuroblasts.

## Conclusions

**Fig. 9:**
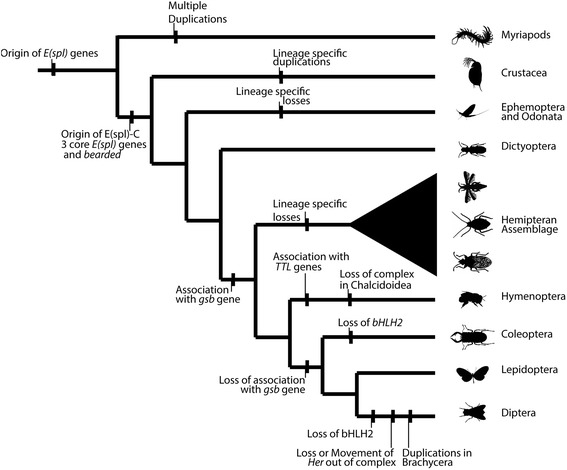
Summary of inferred evolutionary events in the evolution of the E(Spl)-C in insects and Crustacea

The genomes of arthropods contain few evolutionary conserved gene complexes [1], the most well known being the Hox [5], runt [88] and E(spl)-C [10]. The E(spl)-C is restricted to Crustacea and insects, but the key bHLH genes arose before the formation of the complex. The complex appears to have become assembled in the lineage leading to insects and crustaceans, possibly though the association of the bHLH genes (with a long evolutionary history of Notch responsiveness) with the *mα* Brd-class gene (Fig. 9). Presumably the formation of this complex gave some advantage in the regulation or expression of these genes, cementing the structure of the complex. The regulation of this complex in *Drosophila* through chromatin conformation regulators [45, 46], and the suggestion of coordinate regulation [46], may provide an explanation for the conservation of the complex through 540 million years of arthropod evolution. This complex has remained stable in insect genomes, but while gene-loss and duplication has reshaped it in some lineages, its complete absence has only been detected in a group of Chalconid wasps.

The pattern of evolution of the E(spl)-C implies some regulatory reason for the conservation of its structure, perhaps on a par with the well-described coordinate regulation of the Hox complex [5]. Examining the expression and function of these genes in species with variations of the complex, and in deeply branching groups such as myriapods, will provide insight into the reasons behind the conservation of this remarkable gene complex.

## Methods

### Gene identification

BHLHO domain genes and Brd-class genes were identified in arthropod genomes using Blast [69], with orthology assigned using a reciprocal blast best-hit approach. Coding sequences were either extracted from gene prediction sets, or, if such predictions were absent or erroneous, using FGENESH [89] on contigs identified as containing bHLHO sequences using tblastn [69]. Predicted proteins were generated and aligned using CLC Genomics Workbench (http://www.clcbio.com).

Genomic analyses were carried out using CLC Genomics Workbench to visualise the placement of bHLHO genes on scaffolds and contigs. Predicted proteins encoded in these regions were analysed with blastp [69], in the first instance, and HMMER [90] (to identify Brd-class encoding genes).

### Phylogenetics

All phylogenetics were carried out using MrBayes [67] using the WAG model of protein [91] which proved to be the most appropriate model after testing using mixed models. Monte-Carlo Markov chains were run for 1000000 generations with the initial 25 % of trees discarded as burn-in. Consensus trees were visualised with Dendroscope [92] or CLC Genomics Workbench.

### Availability of data and materials

All sequences and accession numbers (either derived from Genbank or the Baylor i5K pilot project (https://www.hgsc.bcm.edu/i5k-pilot-project-summary)) are available in Additional file 2.

### Ethics

This manuscript describes analysis of publically available genome sequences and annotation (used with permission) and thus did not require animal ethics approval.

## Additional files

Additional file 1:
**Table of bHLH Orange domain genes used in this analysis.** (XLSX 35 kb) Additional file 2:
**Alignments used for phylogenetic analyses.** (TXT 111 kb)

## References

[1] Zdobnov EM, Bork P (2007). Quantification of insect genome divergence. Trends Genet.

[2] Barbazuk WB, Korf I, Kadavi C, Heyen J, Tate S, Wun E, Bedell JA, McPherson JD, Johnson SL (2000). The syntenic relationship of the zebrafish and human genomes. Genome Res.

[3] Kohn M, Hogel J, Vogel W, Minich P, Kehrer-Sawatzki H, Graves JA, Hameister H (2006). Reconstruction of a 450-My-old ancestral vertebrate protokaryotype. Trends Genet.

[4] Kikuta H, Laplante M, Navratilova P, Komisarczuk AZ, Engstrom PG, Fredman D, Akalin A, Caccamo M, Sealy I, Howe K (2007). Genomic regulatory blocks encompass multiple neighboring genes and maintain conserved synteny in vertebrates. Genome Res.

[5] Hughes CL, Kaufman TC (2002). Hox genes and the evolution of the arthropod body plan. Evol Dev.

[6] Knust E, Schrons H, Grawe F, Campos-Ortega JA (1992). Seven genes of the Enhancer of split complex of Drosophila melanogaster encode helix-loop-helix proteins. Genetics.

[7] Delidakis C, Monastirioti M, Magadi SS (2014). E(spl): genetic, developmental, and evolutionary aspects of a group of invertebrate Hes proteins with close ties to Notch signaling. Curr Top Dev Biol.

[8] Apidianakis Y, Nagel AC, Chalkiadaki A, Preiss A, Delidakis C (1999). Overexpression of the m4 and malpha genes of the E(spl)-complex antagonizes notch mediated lateral inhibition. Mech Dev.

[9] Lai EC, Bodner R, Posakony JW (2000). The enhancer of split complex of Drosophila includes four Notch-regulated members of the bearded gene family. Development.

[10] Duncan EJ, Dearden PK (2010). Evolution of a genomic regulatory domain: the role of gene co-option and gene duplication in the Enhancer of split complex. Genome Res.

[11] Schrons H, Knust E, Campos-Ortega JA (1991). Toward a functional dissection of the Enhancer-of-split gene complex. J Neurogenet.

[12] Delidakis C, Artavanis-Tsakonas S (1992). The Enhancer of split [E(spl)] locus of Drosophila encodes seven independent helix-loop-helix proteins. Proc Natl Acad Sci U S A.

[13] Schrons H, Knust E, Campos-Ortega JA (1992). The Enhancer of split complex and adjacent genes in the 96 F region of Drosophila melanogaster are required for segregation of neural and epidermal progenitor cells. Genetics.

[14] Oellers N, Tietze K, Knust E (1993). The genes of the Enhancer-of-split complex of Drosophila melanogaster encodes regulatory helix-loop-helix proteins. J Neurogenet.

[15] Lai EC, Posakony JW (1997). The Bearded box, a novel 3′ UTR sequence motif, mediates negative post-transcriptional regulation of Bearded and Enhancer of split Complex gene expression. Development.

[16] Leviten MW, Lai EC, Posakony JW (1997). The Drosophila gene Bearded encodes a novel small protein and shares 3′ UTR sequence motifs with multiple Enhancer of split complex genes. Development.

[17] Welshons WJ (1956). Dosage experiments with split mutants in the presence of an enhancer of split. Drosophila Inf Serv.

[18] Jennings B, Preiss A, Delidakis C, Bray S (1994). The Notch signalling pathway is required for Enhancer of split bHLH protein expression during neurogenesis in the Drosophila embryo. Development.

[19] Knust E (1994). Cell fate choice during early neurogenesis in Drosophila melanogaster. Perspect Dev Neurobiol.

[20] Bray SJ (1997). Expression and function of Enhancer of split bHLH proteins during Drosophila neurogenesis. Perspect Dev Neurobiol.

[21] Hartenstein V, Wodarz A (2013). Initial neurogenesis in Drosophila. Wiley Interdiscipl Rev Dev Biol.

[22] Nakao K, Campos-Ortega JA (1996). Persistent expression of genes of the enhancer of split complex suppresses neural development in Drosophila. Neuron.

[23] Oellers N, Dehio M, Knust E (1994). bHLH proteins encoded by the Enhancer of split complex of Drosophila negatively interfere with transcriptional activation mediated by proneural genes. Mol Gen Genet.

[24] Ruiz-Gomez M, Ghysen A (1993). The expression and role of a proneural gene, achaete, in the development of the larval nervous system of Drosophila. EMBO J.

[25] Tepass U, Hartenstein V (1995). Neurogenic and proneural genes control cell fate specification in the Drosophila endoderm. Development.

[26] Lecourtois M, Schweisguth F (1995). The neurogenic suppressor of hairless DNA-binding protein mediates the transcriptional activation of the enhancer of split complex genes triggered by Notch signaling. Genes Dev.

[27] Gigliani F, Longo F, Gaddini L, Battaglia PA (1996). Interactions among the bHLH domains of the proteins encoded by the Enhancer of split and achaete-scute gene complexes of Drosophila. Mol Gen Genet.

[28] Heitzler P, Bourouis M, Ruel L, Carteret C, Simpson P (1996). Genes of the Enhancer of split and achaete-scute complexes are required for a regulatory loop between Notch and Delta during lateral signalling in Drosophila. Development.

[29] Jennings BH, Tyler DM, Bray SJ (1999). Target specificities of Drosophila enhancer of split basic helix-loop-helix proteins. Mol Cell Biol.

[30] Kaul A, Schuster E, Jennings BH (2014). The Groucho co-repressor is primarily recruited to local target sites in active chromatin to attenuate transcription. PLoS Genet.

[31] Lai EC, Bodner R, Kavaler J, Freschi G, Posakony JW (2000). Antagonism of notch signaling activity by members of a novel protein family encoded by the bearded and enhancer of split gene complexes. Development.

[32] Pavlopoulos E, Pitsouli C, Klueg KM, Muskavitch MA, Moschonas NK, Delidakis C (2001). neuralized Encodes a peripheral membrane protein involved in delta signaling and endocytosis. Dev Cell.

[33] Preiss A, de Celis J, Jennings B, Wech I, Wurmbach E, Delidakis C, Bray S (1997). Distinct expression of individual Enhancer of split genes. J Neurogenet.

[34] Wech I, Bray S, Delidakis C, Preiss A (1999). Distinct expression patterns of different enhancer of split bHLH genes during embryogenesis of Drosophila melanogaster. Dev Genes Evol.

[35] Eastman DS, Slee R, Skoufos E, Bangalore L, Bray S, Delidakis C (1997). Synergy between suppressor of Hairless and Notch in regulation of Enhancer of split m gamma and m delta expression. Mol Cell Biol.

[36] Cave JW, Xia L, Caudy M (2011). Differential regulation of transcription through distinct Suppressor of Hairless DNA binding site architectures during Notch signaling in proneural clusters. Mol Cell Biol.

[37] Nellesen DT, Lai EC, Posakony JW (1999). Discrete enhancer elements mediate selective responsiveness of enhancer of split complex genes to common transcriptional activators. Dev Biol.

[38] Maeder ML, Polansky BJ, Robson BE, Eastman DA (2007). Phylogenetic footprinting analysis in the upstream regulatory regions of the Drosophila enhancer of split genes. Genetics.

[39] Bailey AM, Posakony JW (1995). Suppressor of hairless directly activates transcription of enhancer of split complex genes in response to Notch receptor activity. Genes Dev.

[40] Furukawa T, Kobayakawa Y, Tamura K, Kimura K, Kawaichi M, Tanimura T, Honjo T (1995). Suppressor of hairless, the Drosophila homologue of RBP-J kappa, transactivates the neurogenic gene E(spl)m8. Idengaku Zasshi.

[41] Fortini ME, Artavanis-Tsakonas S (1994). The suppressor of Hairless protein participates in Notch receptor signaling. Cell.

[42] Ungerer P, Eriksson BJ, Stollewerk A (2012). Unravelling the evolution of neural stem cells in arthropods: notch signalling in neural stem cell development in the crustacean Daphnia magna. Dev Biol.

[43] Lai EC, Burks C, Posakony JW (1998). The K box, a conserved 3′ UTR sequence motif, negatively regulates accumulation of enhancer of split complex transcripts. Development.

[44] Lai EC, Tam B, Rubin GM (2005). Pervasive regulation of Drosophila Notch target genes by GY-box-, Brd-box-, and K-box-class microRNAs. Genes Dev.

[45] Schaaf CA, Misulovin Z, Sahota G, Siddiqui AM, Schwartz YB, Kahn TG, Pirrotta V, Gause M, Dorsett D (2009). Regulation of the Drosophila Enhancer of split and invected-engrailed gene complexes by sister chromatid cohesion proteins. PLoS One.

[46] Schaaf CA, Misulovin Z, Gause M, Koenig A, Dorsett D (2013). The Drosophila enhancer of split gene complex: architecture and coordinate regulation by notch, cohesin, and polycomb group proteins. G3.

[47] Ingham PW, Pinchin SM, Howard KR, Ish-Horowicz D (1985). Genetic analysis of the hairy locus of Drosophila melanogaster. Genetics.

[48] Rushlow CA, Hogan A, Pinchin SM, Howe KM, Lardelli M, Ish-Horowicz D (1989). The Drosophila hairy protein acts in both segmentation and bristle patterning and shows homology to N-myc. EMBO J.

[49] Sommer RJ, Tautz D (1993). Involvement of an orthologue of the Drosophila pair-rule gene hairy in segment formation of the short germ-band embryo of Tribolium (Coleoptera). Nature.

[50] Damen WG, Weller M, Tautz D (2000). Expression patterns of *hairy*, *even-skipped*, and *runt* in the spider *Cupiennius salei* imply that these genes were segmentation genes in a basal arthropod. Proc Natl Acad Sci U S A.

[51] Wilson MJ, Dearden PK (2012). Pair-rule gene orthologues have unexpected maternal roles in the honeybee (Apis mellifera). PLoS One.

[52] Bier E, Vaessin H, Younger-Shepherd S, Jan LY, Jan YN (1992). deadpan, an essential pan-neural gene in Drosophila, encodes a helix-loop-helix protein similar to the hairy gene product. Genes Dev.

[53] Younger-Shepherd S, Vassin H, Bier E, Jan LY, Jan YN (1992). deadpan, an essential pan-neural gene encoding an HLH protein, acts as a denominator in Drosophila sex determination. Cell.

[54] San-Juán BP, Baonza A (2011). The bHLH factor deadpan is a direct target of Notch signaling and regulates neuroblast self-renewal in Drosophila. Dev Biol.

[55] Monastirioti M, Giagtzoglou N, Koumbanakis KA, Zacharioudaki E, Deligiannaki M, Wech I, Almeida M, Preiss A, Bray S, Delidakis C (2010). Drosophila Hey is a target of Notch in asymmetric divisions during embryonic and larval neurogenesis. Development.

[56] Kadener S, Stoleru D, McDonald M, Nawathean P, Rosbash M (2007). Clockwork Orange is a transcriptional repressor and a new Drosophila circadian pacemaker component. Genes Dev.

[57] Lim C, Chung BY, Pitman JL, McGill JJ, Pradhan S, Lee J, Keegan KP, Choe J, Allada R (2007). Clockwork orange encodes a transcriptional repressor important for circadian-clock amplitude in Drosophila. Curr Biol.

[58] Matsumoto A, Ukai-Tadenuma M, Yamada RG, Houl J, Uno KD, Kasukawa T, Dauwalder B, Itoh TQ, Takahashi K, Ueda R (2007). A functional genomics strategy reveals clockwork orange as a transcriptional regulator in the Drosophila circadian clock. Genes Dev.

[59] Richier B, Michard-Vanhee C, Lamouroux A, Papin C, Rouyer F (2008). The clockwork orange Drosophila protein functions as both an activator and a repressor of clock gene expression. J Biol Rhythm.

[60] Kobayashi T, Kageyama R (2014). Expression dynamics and functions of Hes factors in development and diseases. Curr Top Dev Biol.

[61] Gazave E, Guillou A, Balavoine G (2014). History of a prolific family: the Hes/Hey-related genes of the annelid Platynereis. EvoDevo.

[62] San Juan BP, Andrade-Zapata I, Baonza A (2012). The bHLH factors Dpn and members of the E(spl) complex mediate the function of Notch signalling regulating cell proliferation during wing disc development. Biol Open.

[63] Geling A, Plessy C, Rastegar S, Strahle U, Bally-Cuif L (2004). Her5 acts as a prepattern factor that blocks neurogenin1 and coe2 expression upstream of Notch to inhibit neurogenesis at the midbrain-hindbrain boundary. Development.

[64] Tannahill D, Bray S, Harris WA (1995). A Drosophila E(spl) gene is “neurogenic” in Xenopus: a green fluorescent protein study. Dev Biol.

[65] i KC (2013). The i5K Initiative: advancing arthropod genomics for knowledge, human health, agriculture, and the environment. J Hered.

[66] Poelchau M, Childers C, Moore G, Tsavatapalli V, Evans J, Lee CY, Lin H, Lin JW, Hackett K (2015). The i5k Workspace@NAL-enabling genomic data access, visualization and curation of arthropod genomes. Nucleic Acids Res.

[67] Ronquist F, Huelsenbeck JP (2003). MrBayes 3: Bayesian phylogenetic inference under mixed models. Bioinformatics.

[68] Chipman AD, Ferrier DE, Brena C, Qu J, Hughes DS, Schroder R, Torres-Oliva M, Znassi N, Jiang H, Almeida FC (2014). The first myriapod genome sequence reveals conservative arthropod gene content and genome organisation in the centipede Strigamia maritima. PLoS Biol.

[69] Altschul S, Gish W, Miller W, Myers E, Lipman D (1990). Basic local alignment search tool. J Mol Biol.

[70] Wurmbach E, Wech I, Preiss A (1999). The Enhancer of split complex of Drosophila melanogaster harbors three classes of Notch responsive genes. Mech Dev.

[71] Schlatter R, Maier D (2005). The Enhancer of split and Achaete-Scute complexes of Drosophilids derived from simple ur-complexes preserved in mosquito and honeybee. BMC Evol Biol.

[72] Chen G, Courey AJ (2000). Groucho/TLE family proteins and transcriptional repression. Gene.

[73] Gutjahr T, Patel NH, Li X, Goodman CS, Noll M (1993). Analysis of the gooseberry locus in Drosophila embryos: gooseberry determines the cuticular pattern and activates gooseberry neuro. Development.

[74] Bhat KM (1996). The patched signaling pathway mediates repression of gooseberry allowing neuroblast specification by wingless during Drosophila neurogenesis. Development.

[75] Duman-Scheel M, Li X, Orlov I, Noll M, Patel NH (1997). Genetic separation of the neural and cuticular patterning functions of gooseberry. Development.

[76] Xue L, Li X, Noll M (2001). Multiple protein functions of paired in Drosophila development and their conservation in the Gooseberry and Pax3 homologs. Development.

[77] Liu W, Xue L (2012). Functional conservation of the Drosophila gooseberry gene and its evolutionary alleles. PLoS One.

[78] Savard J, Marques-Souza H, Aranda M, Tautz D (2006). A segmentation gene in tribolium produces a polycistronic mRNA that codes for multiple conserved peptides. Cell.

[79] Davis GK, Jaramillo CA, Patel NH (2001). Pax group III genes and the evolution of insect pair-rule patterning. Development.

[80] Osborne PW, Dearden PK (2005). Expression of Pax group III genes in the honeybee (Apis mellifera). Dev Genes Evol.

[81] Keller RG, Desplan C, Rosenberg MI (2010). Identification and characterization of Nasonia Pax genes. Insect Mol Biol.

[82] Martinson EO, Wheeler D, Wright J, Mrinalini, Siebert AL, Werren JH (2014). Nasonia vitripennis venom causes targeted gene expression changes in its fly host. Mol Ecol.

[83] Dove H, Stollewerk A (2003). Comparative analysis of neurogenesis in the myriapod Glomeris marginata (Diplopoda) suggests more similarities to chelicerates than to insects. Development.

[84] Brenneis G, Stollewerk A, Scholtz G (2013). Embryonic neurogenesis in Pseudopallene sp. (Arthropoda, Pycnogonida) includes two subsequent phases with similarities to different arthropod groups. Evo Devo.

[85] Stollewerk A, Chipman AD (2006). Neurogenesis in myriapods and chelicerates and its importance for understanding arthropod relationships. Integr Comp Biol.

[86] Kadner D, Stollewerk A (2004). Neurogenesis in the chilopod Lithobius forficatus suggests more similarities to chelicerates than to insects. Dev Genes Evol.

[87] Stollewerk A, Weller M, Tautz D (2001). Neurogenesis in the spider Cupiennius salei. Development.

[88] Duncan EJ, Wilson MJ, Smith JM, Dearden PK (2008). Evolutionary origin and genomic organisation of runt-domain containing genes in arthropods. BMC Genomics.

[89] Solovyev V, Kosarev P, Seledsov I, Vorobyev D (2006). Automatic annotation of eukaryotic genes, pseudogenes and promoters. Genome Biol.

[90] Finn RD, Clements J, Eddy SR (2011). HMMER web server: interactive sequence similarity searching. Nucleic Acids Res.

[91] Whelan S, Goldman N (2001). A general empirical model of protein evolution derived from multiple protein families using a maximum-likelihood approach. Mol Biol Evol.

[92] Huson DH, Richter DC, Rausch C, Dezulian T, Franz M, Rupp R (2007). Dendroscope: an interactive viewer for large phylogenetic trees. BMC Bioinformatics.

